# Recombinant *Bacteroides fragilis* enterotoxin-1 (rBFT-1) promotes proliferation of colorectal cancer via CCL3-related molecular pathways

**DOI:** 10.1515/biol-2021-0043

**Published:** 2021-04-27

**Authors:** Xiaoliang Xie, Dan Jiang, Xuebing Zhou, Xiaoping Ye, Ping Yang, Yaqin He

**Affiliations:** Department of Colorectal Surgery, General Hospital of Ningxia Medical University, Yinchuan, China; School of Clinical Medicine, Ningxia Medical University, Yinchuan, China; Department of Gastriointestinal Surgery, People’s Hospital of Ningxia Hui Autonomous Region, Yinchuan, China; Surgical Department, General Hospital of Ningxia Medical University, Yinchuan, China

**Keywords:** recombinant *Bacteroides fragilis* enterotoxin-1, colorectal cancer, chemokine C–C motif ligand 3, NF-κB

## Abstract

Colorectal cancer (CRC) is one of the most frequently diagnosed cancers worldwide and stands among the leading causes of cancer-related deaths. Although deregulation of the microbiota in the gastrointestinal tract has been frequently described in CRC, very little is known about the precise molecular mechanisms by which bacteria and their toxins modulate the process of tumorigenesis and behavior of cancer cells. In this study, we produced recombinant *Bacteroides fragilis* enterotoxin-1 (rBFT1) and demonstrate that rBFT1 could promote cell proliferation in colorectal cancer cells and accelerate tumor growth *in vivo*. To identify the mechanisms, we further investigated CCL3/CCR5 and NF-κB pathway. We found that CCL3, CCR5, NF-κB, and TRAF-6 were dramatically upregulated after rBFT1 treatment, thus suggesting that the role of rBFT1 in CRC progression may be associated with CCL3/CCR5 and NF-κB pathways. Collectively, our results indicate that rBFT1 serves as a tumor promoter and plays a crucial role in inducing the proliferation of CRC via accelerating CCL3-related molecular pathway, thus giving insights into mechanistic underpinnings for the prevention and treatment of CRC.

## 1 Introduction

Colorectal cancer is the third most prevalent cancer worldwide. It is often associated with change in people’s lifestyle due to improving economic situation and subsequent changes in food habits [1]. Genetic and environmental factors have also been implicated in colorectal cancer [2]. Because there are approximately 3 × 10^13^ bacteria living in and interacting with the colorectum, microorganisms are important for gastrointestinal physiology and are known to play significant roles in intestinal diseases and CRC [3,4]. Recently, the role of gut microbiota in CRC development has gained attention from several researchers [5,6,7]. However, the complexity of gut environment results in difficulties and challenges in the way of clear understanding of the roles and mechanisms of gut microbiota in CRC.

Extensive research has linked the role of gut microbiota to the colorectal cancer carcinogenesis. Compared with the microbiota in healthy individuals, different ecological microenvironments in patients with CRC have been displayed. Other than *Escherichia coli*, *Enterococcus faecalis*, and other bacterium, *Bacteroides fragilis* (BF) which consists of different subtypes had been indicated as one of the major carcinogenic bacteria in the process of colorectal cancer growth [3]. Our previous study found that *Bacteroides fragilis’* number increased significantly in fecal samples of patients with CRC compared with healthy people. Other studies showed that the presence of the *Enterotoxigenic Bacteroides fragilis* (ETBF), which could be detected in 90% of CRC patients and only 50% of healthy individuals, was significantly associated with CRC [8]. *Bacteroides fragilis* toxin (BFT, Fragilysin) encoded by ETBF is considered to be the main virulence factor [9]. BFT consists of three isotypes, including BFT-1, BFT-2, and BFT-3, of which BFT-1 is the most common, accounting for about 75% of all secreted isotypes [8]. BFT is frequently associated with diarrhea in adults and children; however, there have been some reports on its correlation with CRC carcinogenesis [10]. When colonic epithelial cells are exposed to BFT, P38, MAPK and ikk complex are activated leading to the subsequent secretion of CXCL-8 by the colonic epithelial cells. Moreover, activation of tyrosine kinase-dependent NF-κB, mitogen-activated protein kinases (MAPKs), and extracellular signal-related kinases (ECK) is involved in the process and leads to carcinogenesis [11].

Chemokine C–C motif ligand 3 (CCL3) belongs to CCL family and is known as macrophage inflammatory protein. It can be found on the surface of different cells, including macrophages, lymphocytes, and epithelial cells, and has been reported to mediate immune cells cytokine secretion and promote various cells’ aggregation and migration [12]. CCR5 is a receptor of CCL3, and CCL3/CCR5 axis was found to play important roles in the invasion and metastasis of malignant tumors. CCR5 was involved in the development of tumors through the interaction with inflammatory factors and tumor-associated genes to regulate NF-κB signaling pathway [13]. Another study showed that LPS affected neuroinflammatory response via upregulation of CCL3 through NF-κB and MAPKs pathways [14]. However, the interplay between NF-κB and CCL3 in CRC remains to be uncovered.

In the present study, we interrogated the potential roles of recombinant *Bacteroides fragilis* toxin-1 (rBFT-1) in CRC. The abundant colonizing bacteria, *Bacteroides fragilis*, in CRC were explored. With the aid of genetic engineering and other biological technologies, such as enzyme-linked immunosorbent assay (ELISA) and western blotting, roles of rBFT1 representing ETBF in CRC progression and the detailed molecular mechanism were clarified, providing a further understanding for CRC occurrence, development, and therapy.

## 2 Materials and methods

### 2.1 Cell culture

CRC cell line (SW620) and normal colon cell line (NCM460) were purchased from ATCC (American Type Culture Collection, VA, USA). Cells were collected and cultivated at 37°C in an incubator with 5% CO_2_ using Leibovitz’s L-15 and RPMI-1640 medium, respectively, supplemented with 10% fetal bovine serum (FBS), 100 U/mL penicillin, and 100 μg/mL streptomycin (GIBCO, Invitrogen, USA).

### 2.2 Human specimens

Fecal samples were collected from 40 consenting patients randomly chosen from the two groups (Healthy people and CRC patients) to study intestinal microbiota and were analyzed by sequencing the V3–V4 region of 16S ribosomal DNA using the Illumina (San Diego, CA) MiSeq (PE 2  ×  300 bp) platform [15].


**Informed consent:** Informed consent has been obtained from all individuals included in this study.
**Ethical approval:** The research related to human use has been complied with all the relevant national regulations, institutional policies, and in accordance with the tenets of the Helsinki Declaration and has been approved by the Ethics committee of General Hospital of Ningxia Medical University, Yinchuan, China.

### 2.3 Expression and purification of recombinant protein BFT1 (rBFT1)

The amino acid sequence of recombinant BFT-1 protein is derived from NCBI (GenBank: ab026625.1) and its amino acid sequence is as following [16]:
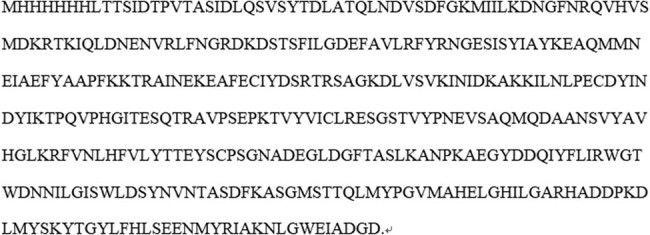


The nucleotides coding BFT1 were obtained by PCR based on the rBFT1 primers (forward and reverse) and were sub-cloned into pET-30a vector to construct the recombinant plasmid. The pET-30a-BFT1 clone strains were cultured on a small scale, induced by IPTG, and screened for the optimal conditions, including IPTG induction concentration, temperature, and time. Then SDS-PAGE was used to determine the best protein expression-inducing conditions and find the location of rBFT1. Then, the positive clone strains were induced on a large scale using optimal-inducing conditions determined in the small-scale batches and rBFT1 was verified as being present in the inclusion bodies. After cell sonication, the culture was centrifuged (20,000 × *g* for 10 min at 4°C; Eppendorf 5702) and sediments were applied to Ni-agarose affinity chromatography. The eluate was collected and examined by SDS-PAGE for rBFT1 expression and verified using anti-His tag antibody by western blot.

### 2.4 Enzyme-linked immunosorbent assay (ELISA)

Human MIP-1α/CCL3 ELISA Kit (BOSTER technology, China) was used to detect the impact of rBFT1 on the expression of CCL3 that SW620 and NCM460 cells secrete in the medium. Manufacturer’s protocol in developing the standard curve was followed. Then, at specified time points (0, 1, 2, 4, 6, 8, 10 and 12 h), the cells’ culture media were centrifuged at 2,000 × *g* for 10 min to remove debris and supernatants were collected and diluted by sample dilution buffer provided with the kit. 100 µL of samples or standard was added to appropriate wells and sealed. The sealed plate was incubated for 90 min at 37°C on a plate shaker set to 400 rpm. Liquid was removed and 100 µL of the anti-CCL3 antibody was added to each well. The plate was sealed and incubated for 60 min at 37°C on a plate shaker set to 400 rpm. Following the incubations, each well was washed with 3× 350 µL wash buffer and after last wash the plate was inverted and blotted against clean paper towels to remove excess liquid. 100 µL of the prepared ABC work solution was added to the plate, sealed, and incubated for 30 min. Then, each well was washed with 5× 350 µL wash buffer and after last wash, the plate was inverted and blotted against clean paper towels to remove excess liquid. Next, 100 µL of TMB substrate was added to each well and incubated for 20 min in the dark on a plate shaker set to 400 rpm. 100 µL of stop solution was added to each well after the incubation period, mixed using a plate shaker for 1 min. Finally, the OD was recorded at 450 nm using an ELISA reader (Bio-Rad Laboratories, Richmond, CA, USA).

### 2.5 Western blots

Cells were cultured or treated by rBFT1, followed by harvesting at predetermined time points. After extensive washing, cells were lysed using Whole Cell Lysis Assay (KeyGEN Biotech, China) according to the manufacturer’s instructions and proteins from the lyste were harvested by centrifugation at 12,000 × *g* for 5 min at 4°C. Protein concentrations were determined by the BCA Assay kit (KeyGEN Biotech, China). Total protein (42 kDa) from each lysate was then separated by SDS-PAGE and subsequently transferred onto PVDF membranes (Millipore, MA, USA). Afterwards, the membranes were blocked with 5% non-fat milk in TBST at room temperature for 1 h, followed by incubation with antibodies against protein of interest overnight at 4°C. The antibodies used in this study included anti-NF-κB/P65 (1:100 in PBS), anti-Macrophage Inflammatory Protein 1α/CCL3 (100 μg/mL in PBS), anti-CCR5 (1:100 in PBS), anti-PI 3 Kinase/P85α (1:200 in PBS), and anti-TRAF6 (1 µg/mL in PBS) (Abcam, CA, USA). After washing with TBST, membranes were striped with appropriate HRP-conjugated secondary antibodies (1:5,000, Abcam, CA, USA) for 1 h at room temperature, followed by visualization using the enhanced Western Bright ECL reagents (Cell Signaling Technology, USA). Protein bands were scanned and analyzed by a chemiluminescence detection system (Bio-Rad, USA).

### 2.6 Cell proliferation assays

The proliferation of SW620 and NCM460 cells after the stimulation with rBFT1 was determined by Cell Counting Kit-8 (CCK-8) assay according to the manufacturer’s instructions using Cell Counting Kit-8 (Dojinodo, Shanghai, China). In brief, cells were plated in 96-well microplates and treated by different concentrations of rBFT1 (100, 500, 1,000 ng/mL) according to the designed groups. Cell proliferation was measured at 0, 1, 2, 6, and 12 h after treatment. For detection, the CCK-8 solution (10 μL) was added to each well and incubated for additional 1.5 h. Absorbance at 450 nm was measured using an ELISA reader (Bio-Rad Laboratories, Richmond, CA, USA). 5 × 10^5^ SW620 or NCM460 cells were seeded into a 24 ­well plate with a total volume of 50 μL of medium per well. The cells were incubated with 5% CO_2_ at 37°C for 24 h. Then, rBFT1 was added into the medium to a final concentration of 1,000 ng/mL. Morphological changes were then observed by optical microscopy at 0, 6, and 12 h, respectively.

### 2.7 Subcutaneous tumorigenicity

For the subcutaneous tumorigenicity assay, 1 × 10^7^ SW620 cells in 200 μL normal saline were inoculated subcutaneously in mice to form tumors. After a week of adaptive assays, high-dose toxin (200 μg/kg), low-dose toxin (100 μg/kg), and blank control (normal saline) were determined to be involved in the assays. Twelve female BALB/c mice were divided into 3 groups, and CRC tumor models were constructed, followed by injection with different dose of rBFT1 every day for 20 days. The general conditions of the mice were observed during the experiment. Tumor volumes and body weights of the mice were measured and recorded every 5 days. At the end of 20 days, the mice were sacrificed by cervical dislocation and the tumor weights and volumes were finally measured.


**Ethical approval:** The research related to animal use has been complied with all the relevant national regulations and institutional policies for the care and use of animals.

### 2.8 Statistical analysis

Data were expressed as the mean ± standard error. One-way analysis of variance, *t*-test, and non-parametric test were used to conduct group comparisons. Categorical variables were compared using Chi-squared test. SPSS 22.0 and GraphPad Prism 5.0 were used. *P* < 0.05 was considered to be indicated as statistically significant difference.

## 3 Results

### 3.1 *Bacteroides fragilis* is abundant in the fecal samples of colorectal carcinoma patients

The microbiota in the gastrointestinal tract has been reported to be involved in CRC progression. In our previous studies, bacterial DNA extracted from fecal samples of 40 CRC patients before (non-surgery) and after surgery (surgery) was screened by 16S rRNA gene sequencing and *Bacteroides fragilis’* number was found to be elevated in CRC patients and decrease after surgery [15].

### 3.2 Recombinant *Bacteroides fragilis* toxin-1 (rBFT-1) promoted the proliferation and growth of CRC *in vitro* and *in vivo*

**Figure 1:**
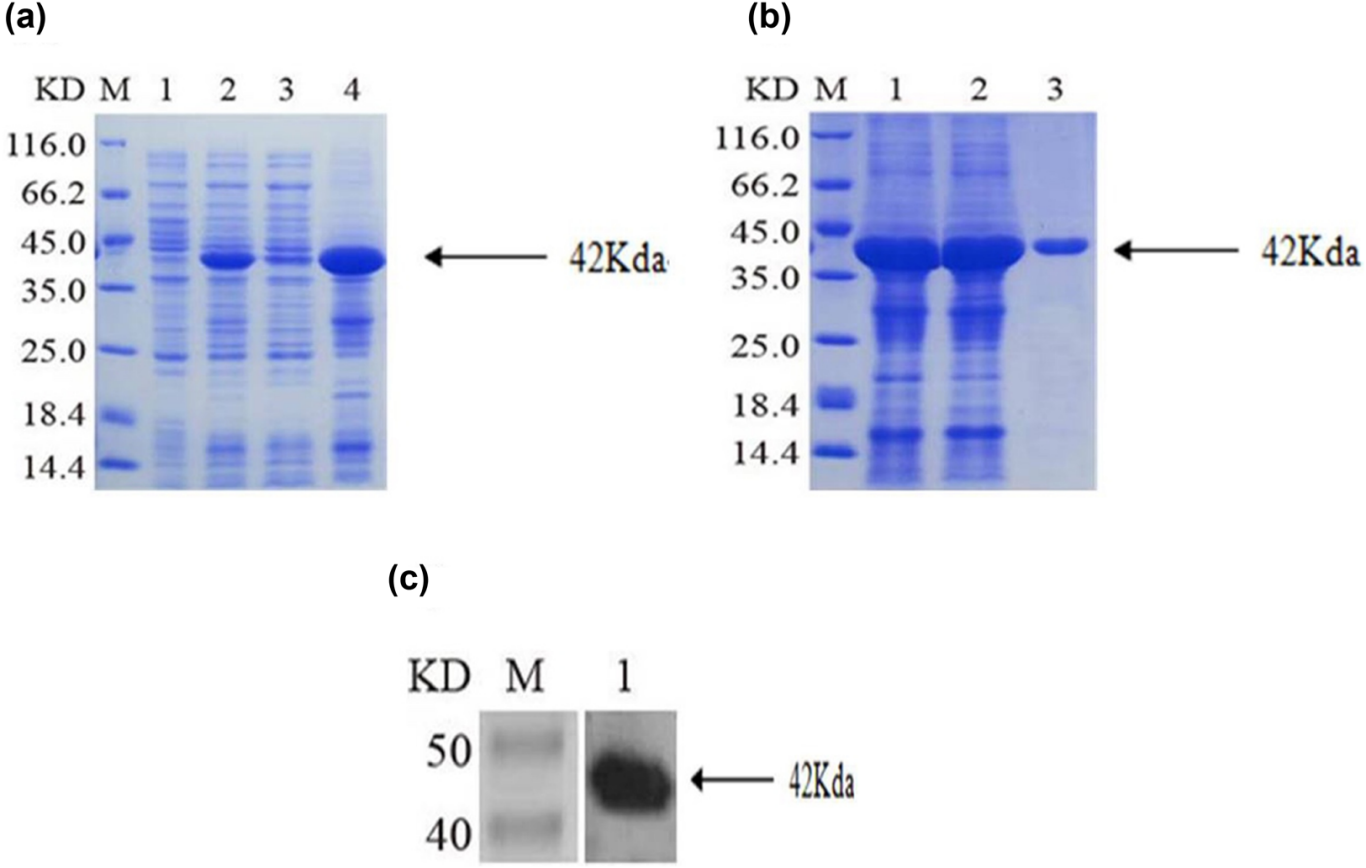
Acquisition, purification, and validation of recombinant BFT1 (rBFT1) protein. (a) Positive expression clone strains could produce rBFT1 (M: Protein marker, 1: Proteins in positive colon strains without induction, 2: Proteins in positive colon strains with IPTG induction, 3: Proteins in supernatants of IPTG-induced positive clone strains after sonication, 4: Proteins in sediments of IPTG-induced positive clone strains after sonication). (b) Ni-agarose affinity chromatography was applied to purify the recombinant BFT1. (M: Protein marker, 1: Total protein, 2: Washing solution, 3: Eluate). (c) The expression of rBFT1 was verified by western blots using anti-His tag antibody.

*Enterotoxigenic Bacteroides fragilis* (ETBF) could secrete *Bacteroides fragilis* toxin (BFT) so as to participate in the process of colorectal diseases. Especially, the mechanism of BFT-1, a known and significant contributor to CRC development, is still unclear. In this study, we produced recombinant BFT-1 to investigate its mechanism in CRC development. As shown in Figure 1a and b, His-tagged recombinant BFT1 protein with a molecular weight of 42 kDa was produced through recombinant expression plasmids construction and IPTG induction, and then purified by Ni-chelating affinity chromatography. Presence of the protein was also verified by western blott assays with anti-His antibodies (Figure 1c).

**Figure 2:**
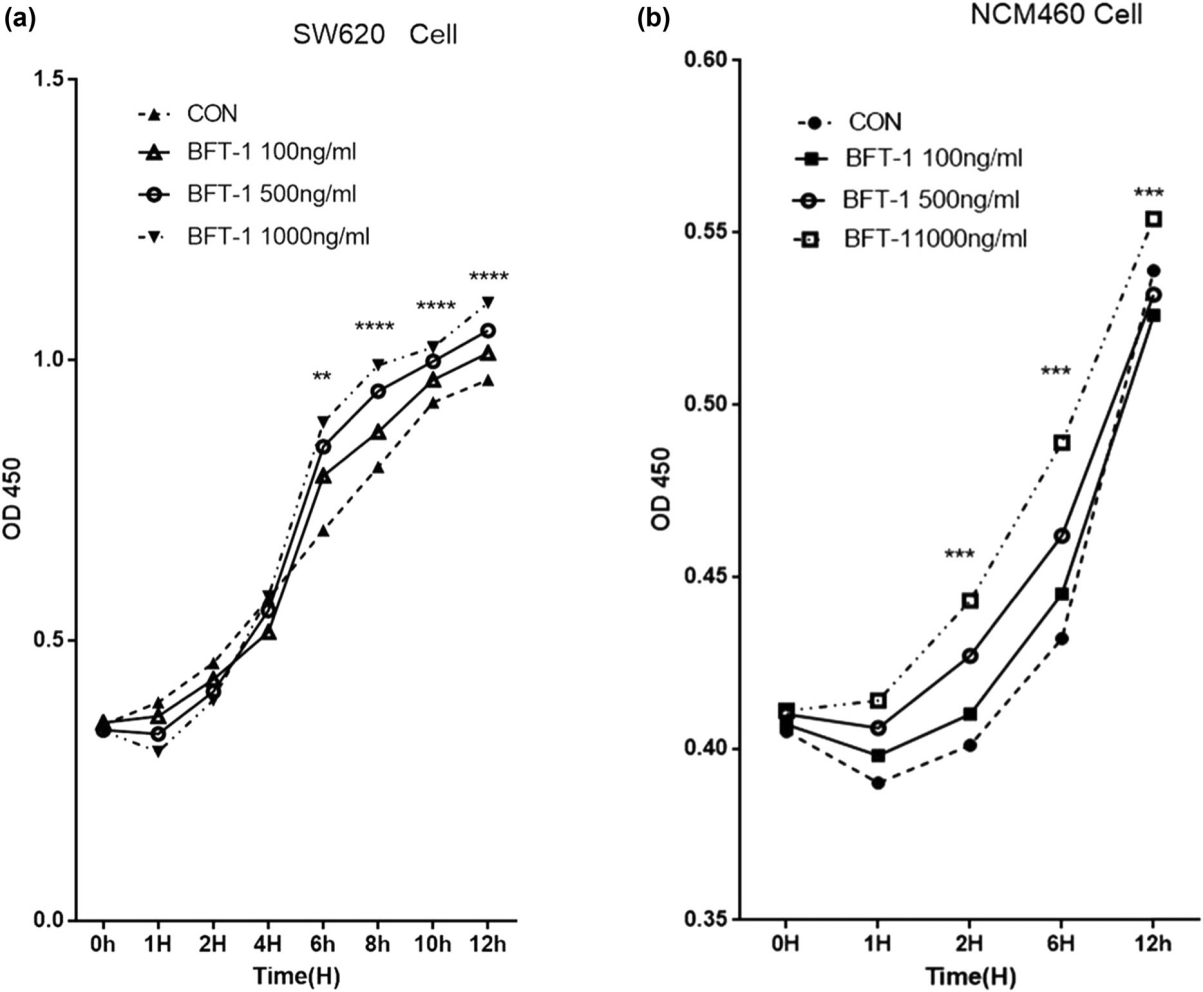
CCK8 assays indicated the impact of rBFT-1 with different concentrations on the proliferation of CRC cells. (a) SW620; (b) NCM460. ns, *, **, and *** represent *P* > 0.05, *P* < 0.05, *P* < 0.01, and *P* < 0.001 respectively.

**Figure 3:**
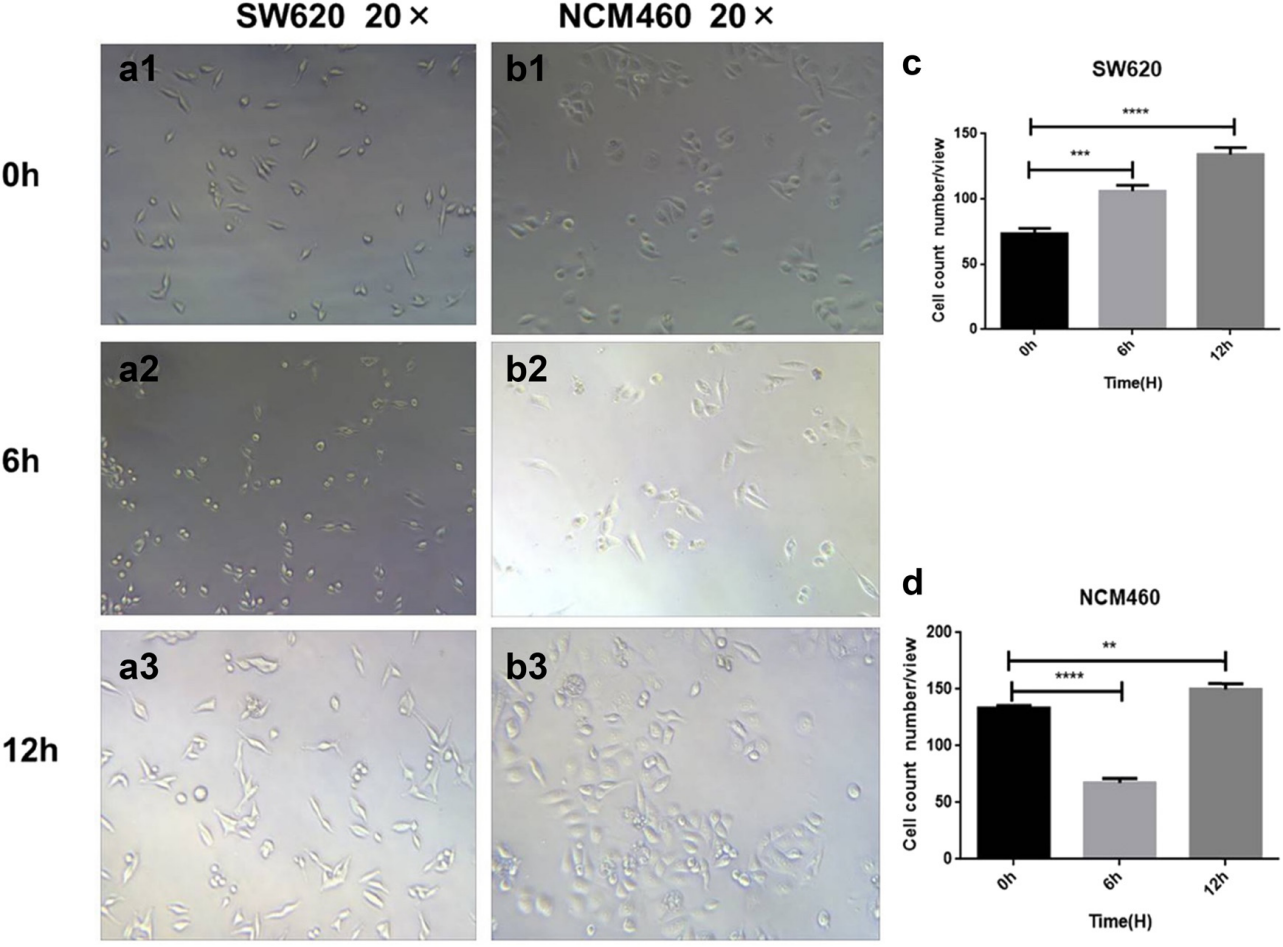
Images and statistical analysis of SW620 and NCM460 (b1–b3) cells stimulated by rBFT-1 for different time; (a1–a3 and c) SW620. (b1–b3 and d). NCM460. ns, *, **, and *** represent *P* > 0.05, *P* < 0.05, *P* < 0.01, and *P* < 0.001, respectively.

**Figure 4:**
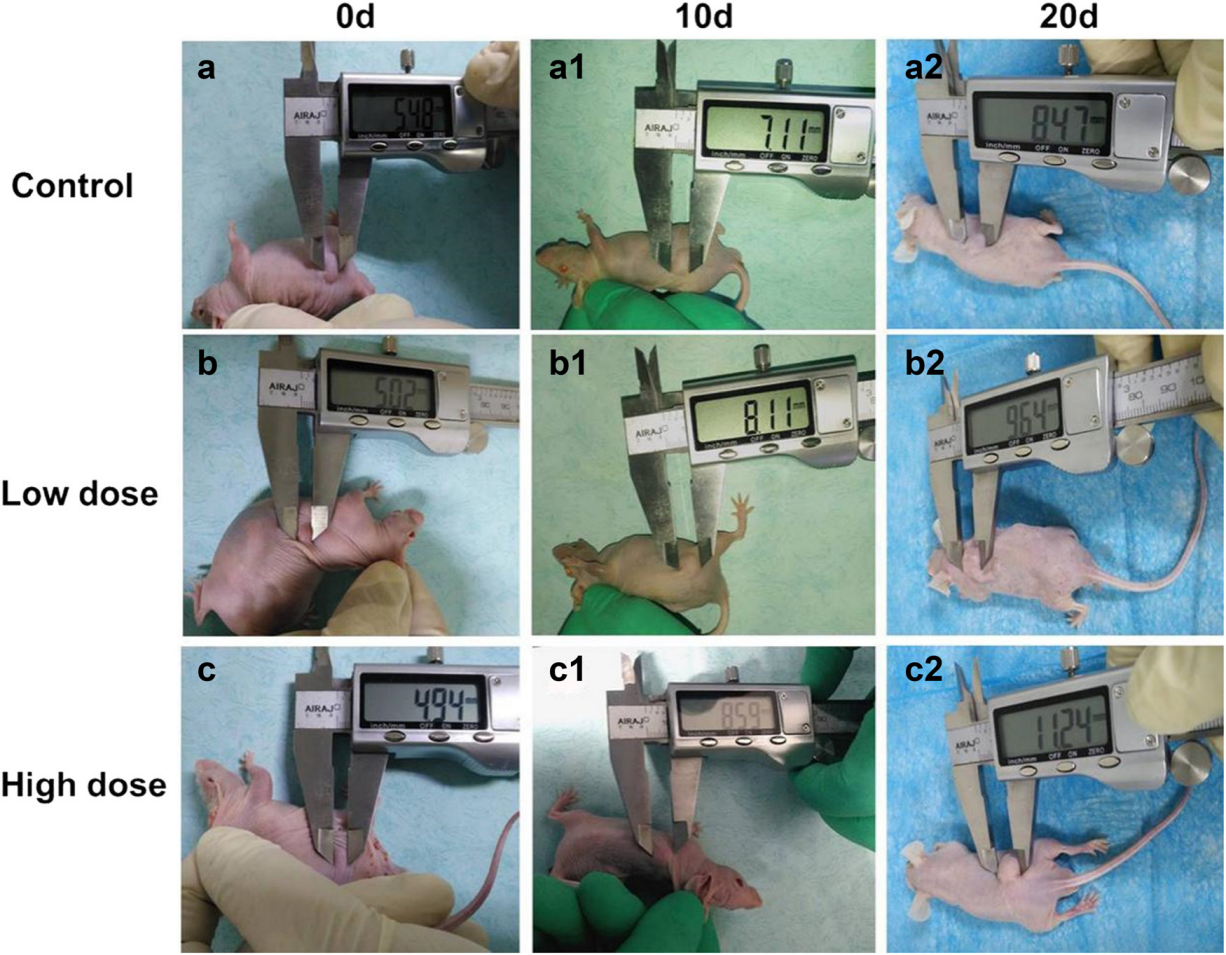
Measurement of tumor diameter after peritumoral injection of different doses of rBFT-1. (a–a2) control group (no BFT group); (b–b2) Low-dose group (100 μg/kg); (c–c2) high-dose group (200 μg/kg).

**Figure 5:**
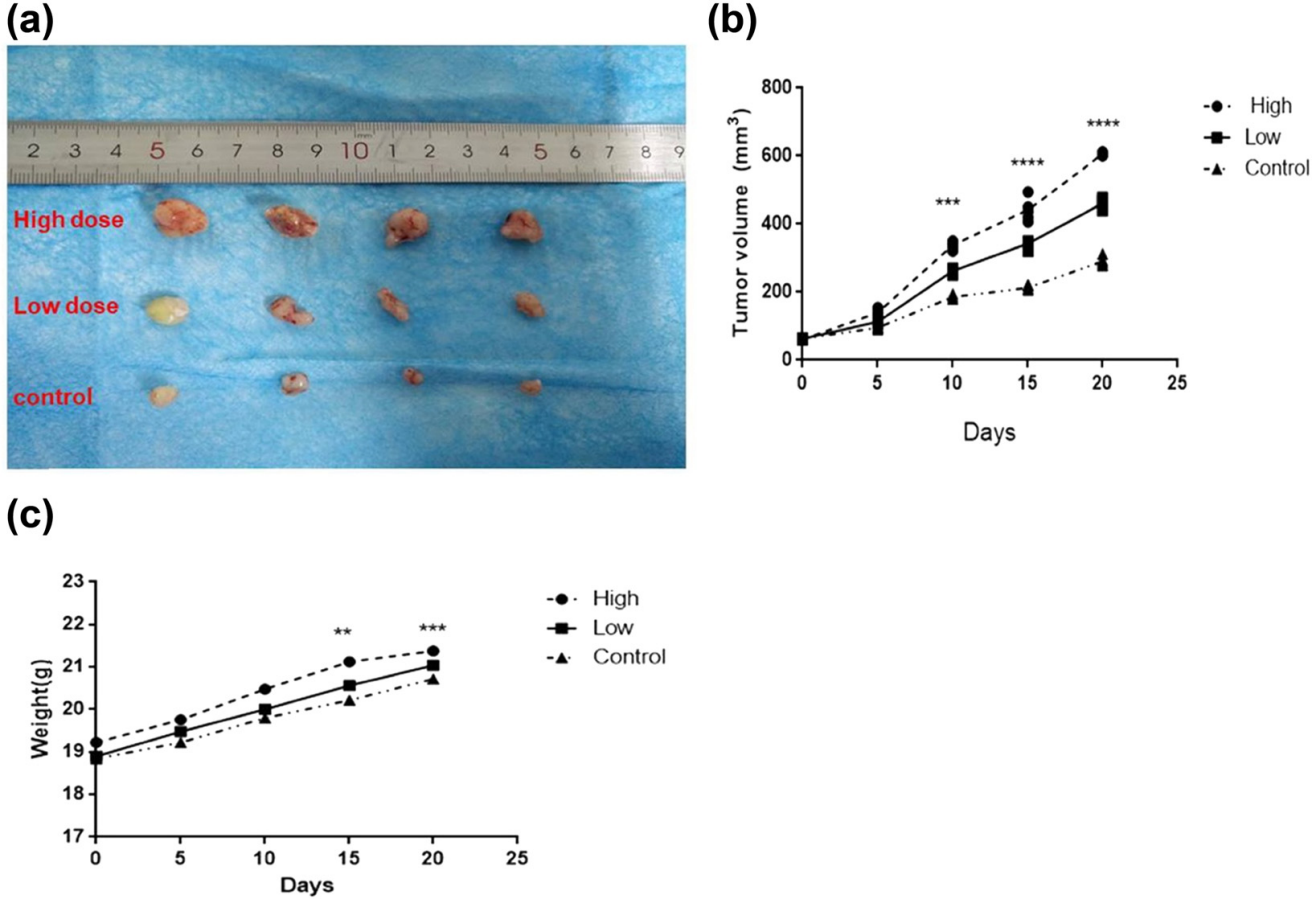
Image showing changes of tumor volumes and body weights after peritumoral injection by rBFT-1 protein on nude mice in different times. (a) Tumor size; (b) tumor volume; (c) tumor weight. High: 200 μg/kg; Low: 100 μg/kg; ns, *, **, and *** represent *P* > 0.05, *P* < 0.05, *P* < 0.01, and *P* < 0.001, respectively.

Recombinant BFT-1 proteins in varying concentrations (100, 500, 1,000 ng/mL) were used to explore its effect on the proliferation of colorectal cells. Consistent with the changes of viewed cells’ shape and number, CCK-8 assays showed that the proliferation of both SW620 and NCM460 cells was evidently promoted in a concentration-dependent way. However, when the stimulation persisted for 12 h, the suppression of rBFT-1 at 1,000 ng/mL was weakened (Figures 2 and 3). To further investigate BFT’s effect on CRC progression, rBFT1 with the respective concentrations of 200 or 100 μg/kg was injected subcutaneously around the colorectal tumor constructed through inoculating SW620 cells into nude mice. As shown in Figures 4 and 5, the high-dose group has higher pace of tumor growth and tumor volume/weight, which suggested that BFT-1 could significantly promote the CRC development.

### 3.3 CCL3, CCR5, NF-κB-related genes expressed higher in CRC cell lines

**Figure 6:**
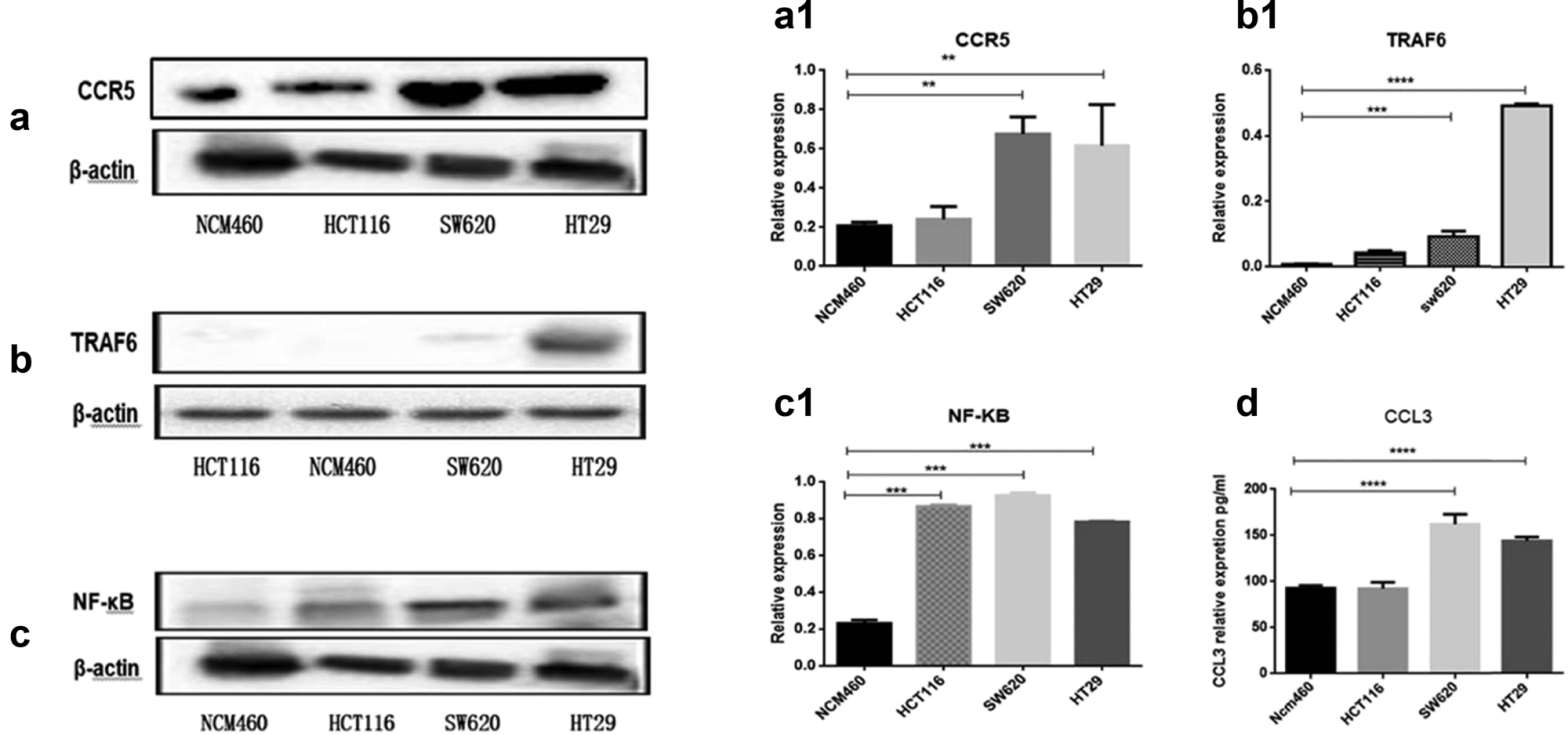
Expression of CCL3, CCR5, NF-κB, and TRAF6 in colorectal and CRC cells was measured by ELISA and Western blots. (a, a1; b, b1; c, c1) Representative immunoblots of CCR5, TRAF6, and NF-κB proteins in three human colon cancer cell lines (HCT116, SW620, HT29) and one normal colonic epithelial cell line (NCM460) (top panel), and the relative levels of these proteins (bottom panel). β-Actin was used as an internal control. (d) The expression of CCL3 protein was determined by ELISA. ns, *, **, and *** represent *P* > 0.05, *P* < 0.05, *P* < 0.01, and *P* < 0.001, respectively.

To explore the roles of CCL3/CCR5 and NF-κB pathways in ETBF-dependent CRC progression, detection of the expression of these associated genes in CRC cell lines (SW620, HT29, and HCT116) and normal colonic epithelial cell line (NCM460) was necessary. The results showed that compared to NCM460 cells, the expression level of CCL3, CCR5, NF-κB, and TRAF6 proteins was elevated in CRC cells (Figure 6). Among that, compared to control, there was an upregulation of CCL3 and CCR5 expression in SW620 and HT29 lines, NF-κB in all of the three CRC cell lines, while TRAF6 was overexpressed only in HT29 cells, indicating CCL3/CCR5 and NF-κB-related pathways may be closely related to CRC progression.

### 3.4 Recombinant BFT1 promotes CRC development in a CCL3/CCR5-dependent way

**Figure 7:**
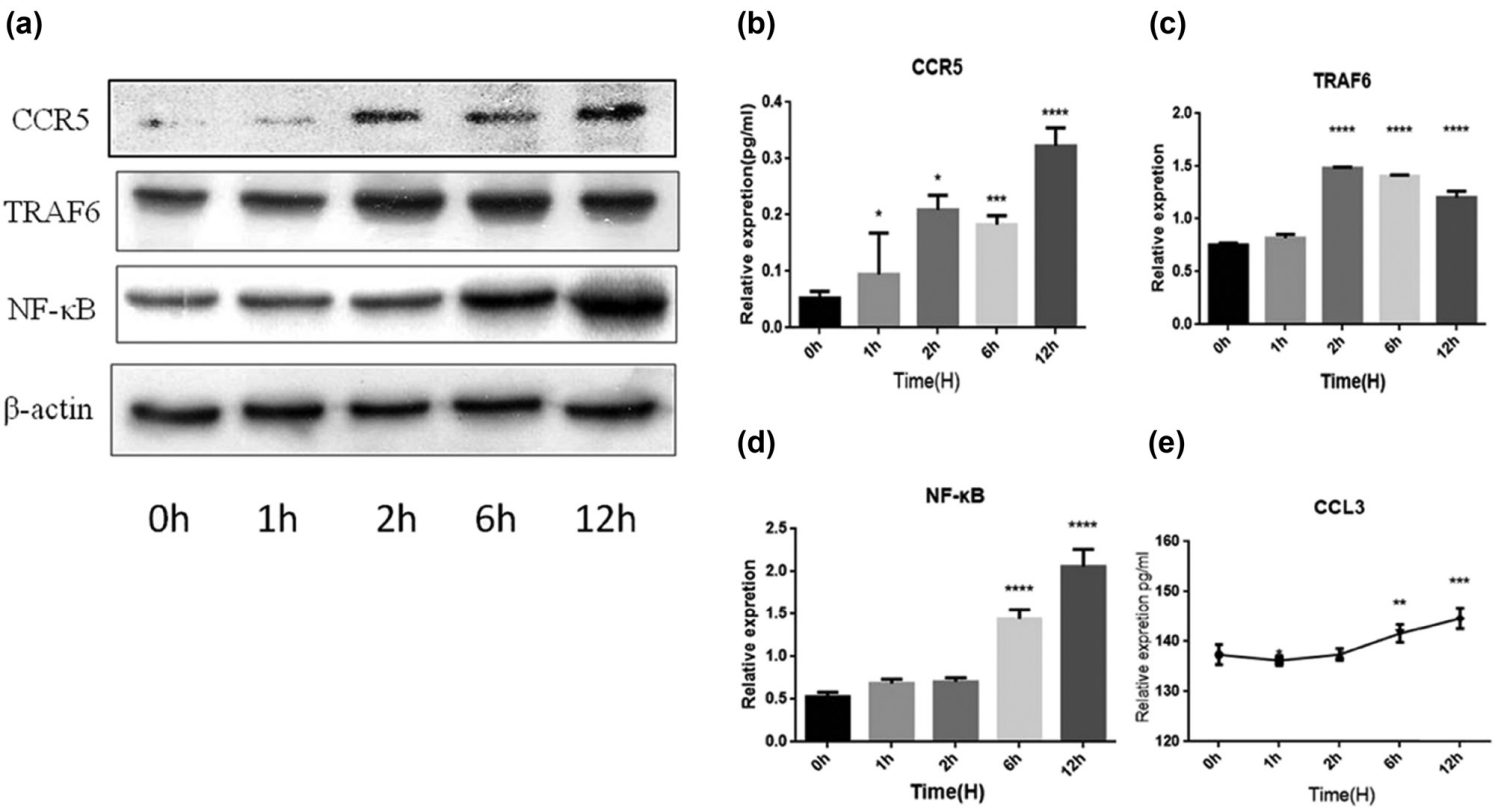
Interference with rBFT-1 has effects on the expression of CCR5, TRAF6, NF-κb, and CCL3 in SW620 cells. (a–d) The relative expression of CCR5, NF-κb, and TRAF6. (e) The relative expression of CCL3 was determined by ELISA. ns, *, **, and *** represent *P* > 0.05, *P* < 0.05, *P* < 0.01, and *P* < 0.001, respectively.

**Figure 8:**
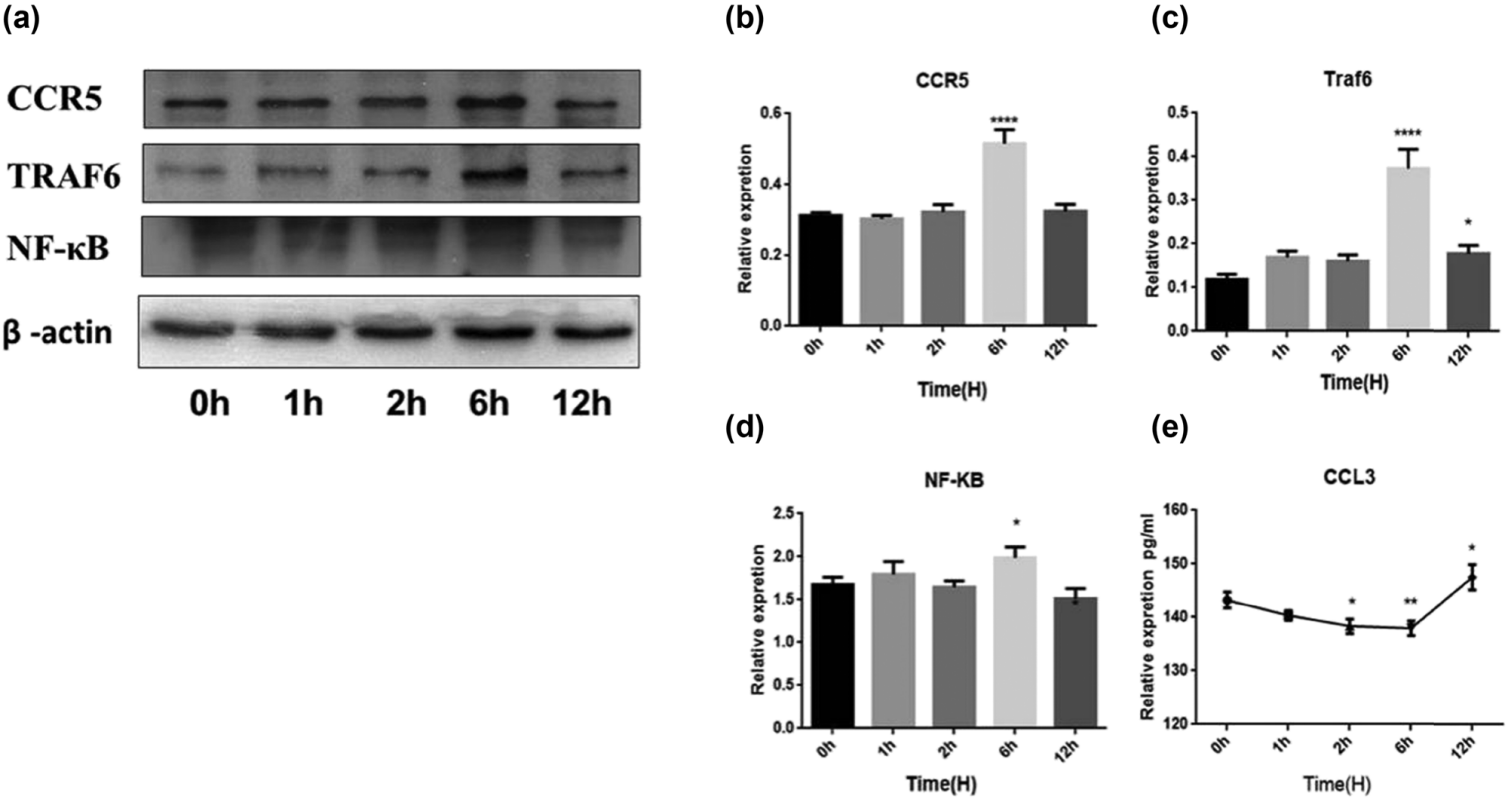
Interference with rBFT-1 has effects on the expression of CCR5, TRAF6, NF-κB, and CCL3 in NCM460 cells. (a–d) The relative expression of CCR5, NF-κb, and TRAF6. (e) The relative expression of CCL3 was determined by ELISA. ns, *, **, and *** represent *P* > 0.05, *P* < 0.05, *P* < 0.01, and *P* < 0.001, respectively.

Given the importance of CCL3 and CCR5 in cancer progression, we tested the impression of BFT-1 on the expression of CCR-5/CCL3, TRAF6/NF-κB. As Figures 7 and 8 showed, rBFT1 was used to stimulate SW620 and NCM460 cells, and the expression of CCL3, CCR5, TRAF6, and NF-κB was measured at 0, 1, 2, 6, and 12 h, respectively. In SW620 cells, after stimulation with rBFT1, the expression of CCL3 slightly decreased at 1 h and 2 h, and then increased after 6 h. The expression of CCR5, TRAF6, and NF-κB increased gradually from 0 to 12 h. Correspondingly, when NCM460 cells were stimulated, the secretion of CCL3 decreased significantly at 1, 2, and 6 h and increased at 12 h. The expression of CCR5, NF-κB, and TRAF6 was also upregulated by the stimulation of rBFT1. Taken together, these results show that rBFT-1 could have effect on the secretion of CCL3, possibly through the CCR5 and NF-κB pathways.

## 4 Discussion

Trillions of bacteria exist in the colon and they are generally separated from the colonic epithelium by a dense mucus layer, which promotes tolerance to foreign antigens by limiting bacterial and epithelial cell contact and inflammatory responses [17]. Strong evidence supports the carcinogenic potential of bacteria that breaches into the colonic mucus layer. Our research suggests that *E. coli* and *B. fragilis* are the two dominant and persistent colonizers in the CRC patients’ fecal and colonic mucosal samples [18]. ETBF can induce colon tumorigenesis in APC^Min/+^ mice and human epidemiological studies showed it is associated with inflammatory bowel disease and sporadic CRC [19]. Furthermore, the role of ETBF to induce CRC is now gaining importance, and BFT has been found to be a critical mediator of ETBF. BFT1 is the major isotype of BFT in fecal samples of CRC patients; thus, we produced rBFT1 by genetic engineering technology and treated it as the representative of ETBF, and further explored the role of ETBF in CRC. Similar to other BFT-CRC-associated studies, we found rBFT1 could promote CRC SW620 cell proliferation. Besides, rBFT1 has similar influence on normal colonic epithelial cells NCM460, implying that BFT may induce transition from normal colonic epithelial cells to tumor cells.

There are a few studies exploring the role of ETBF and BFT1 in the promotion of CRC development. Liam Chung reported that in ETBF-associated carcinogenesis, IL17 combined with receptors on the surface of colonic epithelial cells, and secretion of chemokine CXC subfamily was elevated via activation of NF-κB pathways [10]. In our study, when rBFT1 was used to stimulate SW620 and NCM460, the secretion of CCL3 displayed a positive relationship with its impression on the proliferation, directing our focus to the CCL3-associated pathways.

CCL3 has been reported to play a role in the proliferation, migration, and invasion of malignant tumor cells [20,21]. Recent studies found that upregulation of CCL3 induces invasion and migration of human lung cancer cells A549 [22]. In C57BL/6 (wild type-wt) mice with chemically induced oral squamous cell carcinoma, the expression of both CCL3 and CCR5 increased, and suppression of CCL3 was shown to reduce the proliferation of tumor cells [23]. As one of the receptors of CCL3, CCR5 was regarded to be able to interact with inflammatory factors and tumor-associated genes to regulate NF-κB pathway participating in the development of cancer. Maraviroc, a CCR5 inhibitor, inhibits murine colon carcinoma cells CT26 and human-derived transplant’s growth and CCL3 expression [24]. Given that CCL3 expression in SW620 and NCM460 cells could be affected by BFT1, we further detected CCR5 expression. Interestingly, CCR5 was significantly upregulated in both SW620 and NCM460 cells after the treatment with rBFT1, indicating that CCL3 and its receptor CCR5 may mediate the roles of rBFT1 or ETBF in the proliferation of CRC. Additionally, NF-κB and TRAF-6 which had been pointed to be related to CCL3/CCR5 were measured, and the results showed they were also affected by rBFT1 and have relationships with changes of CCL3 and CCR5. Without a doubt, rBFT-1 presents different biological characters than wild type BFT-1, and it might participate in the process of CRC through more complex genetic network. Therefore, the detailed mechanisms in which ETBF promotes CRC development need further investigation. However, rBFT1 promotes proliferation of colorectal cancer via CCL3-related pathway stimulation, providing an insight to the mechanisms of ETBF’s effect on CRC tumorigenesis and brings out a new perspective on prevention and therapy ways of CRC by employing an intestinal mucosal vaccine.
